# Neuroticism and conscientiousness respectively positively and negatively correlated with the network characteristic path length in dorsal lateral prefrontal cortex: A resting‐state fNIRS study

**DOI:** 10.1002/brb3.1074

**Published:** 2018-07-27

**Authors:** Meng‐Yun Wang, Juan Zhang, Feng‐Mei Lu, Yu‐Tao Xiang, Zhen Yuan

**Affiliations:** ^1^ Faculty of Health Sciences University of Macau Taipa China; ^2^ Faculty of Education University of Macau Taipa China; ^3^ Chengdu Mental Health Center Chengdu China; ^4^ MOE Key Lab for Neuroinformation The Clinical Hospital of Chengdu Brain Science Institute University of Electronic Science and Technology of China Chengdu China

**Keywords:** big five personality traits, conscientiousness, fNIRS, graph theory, neuroticism, small world

## Abstract

**Background:**

Accumulating evidence shows that the dorsal lateral prefrontal cortex (dlPFC) is implicated in personality traits. In this study, resting‐state functional near infrared spectroscopy (fNIRS) combined with small‐world analysis was utilized to examine the relationship between the network properties of dlPFC and personality traits.

**Methods:**

Thirty college students (aged between 20 and 29) were recruited from the University of Macau campus, whose personality scores were accessed with the NEO‐FFT questionnaire. Graph theory combined with resting‐state fNIRS data was used to quantify the network properties of dlPFC, whereas Pearson correlation analysis was performed to generate the relationship between the small‐world indicators and personality scores.

**Results:**

Compared to matched random networks, the resting‐state brain networks exhibited a larger clustering coefficient (*C*_*p*_, 0.1–0.66), shorter characteristic path length (*L*_*p*_, 0.1–0.66), and higher global (*E*_*g*_, 0.1–0.66) and local efficiency (*E*_loc_, 0.1–0.65). In particular, conscientiousness (*r *=* *−0.63) and neuroticism (*r *=* *0.40) respectively showed negative and positive correlation with the *L*_*p*_.

**Conclusions:**

The resting‐state functional brain networks in dlPFC exhibited the small‐world properties. In addition, participants with higher conscientiousness scores showed a shorter *L*_*p*_.

## 1 INTRODUCTION

Human brain is a complex and dynamic system (Sporns, 2014), which organizes and controls individuals’ interaction with the environment. The frontal lobe, especially the prefrontal cortex (PFC) plays an essential role in various high‐level cognitive functions, such as executive functions (Mansouri, Tanaka, & Buckley, 2009; Miller, 2000; Miller & Cohen, 2001), reasoning and planning (Wood & Grafman, 2003), decision making (Wallis, 2007), social cognition, and moral judgment (Forbes & Grafman, 2010). Meanwhile, the deficits in PFC functions are involved in the pathophysiology of several psychiatric and neurological disorders such as schizophrenia, drug addiction, mood disorders, and Alzheimer's disease (Fuster, 2001; Goto, Yang, & Otani, 2010). Therefore, inspecting the PFC's organizing patterns is not only crucial for us to elucidate the complex neural mechanism of high‐level cognitive functions or brain disorders, but also absolutely necessary to pave a way for new treatments.

Intriguingly, previous reports have demonstrated that the structures and functions of PFC are strongly correlated with personality traits (DeYoung et al., 2010; Kennis, Rademaker, & Geuze, 2013), which consist of five factors such as openness, conscientiousness, extraversion, agreeable, and neuroticism (Costa & McCrae, 1992). Regarding the relationship between structural features of PFC and personality traits, past reports highlighted that conscientiousness and neuroticism are relevant with the lateral PFC volumes (DeYoung et al., 2010). For example, individuals with high scores of conscientiousness or neuroticism exhibit a large or small dorsal lateral PFC (dlPFC) volume, respectively (DeYoung et al., 2010; Kapogiannis, Sutin, Davatzikos, Costa, & Resnick, 2013; Wright et al., 2006). In particular, a recent lesion study revealed that the focal damage to the left dlPFC showed significant correlation with high neuroticism and low conscientious scores (Forbes et al., 2014). In addition, the relationships between the PFC functioning and personality traits have also been explored by task‐state studies, such as working memory. It was discovered that the dlPFC activation changes were positively correlated with extraversion (Kumari, ffytche, Williams, & Gray, 2004). Another interesting report demonstrated that the brain activation in left frontal cortical regions associated with negative pictures showed positive correlation with participants’ neuroticism scores (Canli et al., 2001). In an additional emotion study, participants were instructed to attempt decreasing emotional responses while viewing moral violation pictures, in which it was observed that the activation regarding voluntary emotion regulation in dlPFC was positively related to neuroticism (Harenski, Kim, & Hamann, 2009).

To date, although extensive studies have been performed to inspect the relationship based on task‐related PFC activation, the correlation between the PFC functioning at rest and personality traits have not been examined. More importantly, enhanced resting‐state brain activities can provide us unique and exclusive information associated with brain cognition and disorders (Biswal, Zerrin Yetkin, Haughton, & Hyde, 1995; Biswal et al., 2010; Lu et al., 2015; Lu, Liu, et al. 2017). In particular, the resting‐state brain networks can be generated by using graph theory (Bullmore & Sporns, 2009; He & Evans, 2010), in which the topological features of our brain's organization such as small‐world network properties (Achard, Salvador, Whitcher, Suckling, & Bullmore, 2006) are quantified.

The aim of this study is to examine the relationship between the small‐world network properties in dlPFC and personality traits. The small world analysis was first proposed in 1998 (Watts & Strogatz, 1998), which now has been widely adopted to characterize complex networks (Bassett & Bullmore, 2006; Bassett & Sporns, 2017; Boccaletti, Latora, Moreno, Chavez, & Hwang, 2006) in different fields, such as airport networks, biological networks and brain networks. In small‐world network analysis, the clustering coefficient of network *C*_*p*_ denotes the local efficiency in information transfer of the network, whereas the characteristic path length *L*_*p*_ (Watts & Strogatz, 1998) describes the global efficiency and the ability of parallel information transmission of the network. In addition, the global and local efficiency (*E*_glob_, *E*_loc_) measure the ability of information transmission of the network (Latora & Marchiori, 2001; Wang et al., 2009). Further, network hubs are referred as those nodes, which are positioned to make strong contributions to global network function and are able to be measured by node degree (*K*) in graph theory (van den Heuvel & Sporns, 2013). The mean *K* represents the network density, in which the network connections are sparse when the average node degree is small. Meanwhile, functional near‐infrared spectroscopy (fNIRS) is a noninvasive and affordable neuroimaging technique (Ehlis, Schneider, Dresler, & Fallgatter, 2014; Ferrari & Quaresima, 2012; Vanderwert & Nelson, 2014), which utilize the near‐infrared light (wavelengths between 680–950 nm) to inspect the brain activation by measuring the concentration changes of oxygenated hemoglobin (HbO) and deoxygenated hemoglobin (HbR) (Ferrari & Quaresima, 2012; Jobsis, 1977; Villringer & Chance, 1997). fNIRS studies have been conducted to reveal the neural mechanisms underlying various cognitive tasks (He, Wang, Li, & Yuan, 2017; Lu, Wang, Zhang, Chen, & Yuan, 2017; Wang, Lu, Hu, Zhang, & Yuan, 2018). However, little is performed to use resting‐state fNIRS to decode the organizational characters of brains (Niu & He, 2014; Niu, Wang, Zhao, Shu, & He, 2012).

In this study, resting‐state fNIRS combined with small‐world network analysis was used to extract the attribute features of functional brain networks associated with personality traits. Since neuroticism/conscientiousness exhibits the relationship with the structure and function of PFC (Canli et al., 2001; DeYoung et al., 2010; Harenski et al., 2009; Kennis et al., 2013), it is expected that neuroticism/conscientiousness might be strongly correlated with the small‐world network indicators in dlPFC at rest as well. It is anticipated that by investigating into this relationship, this study can help to further reveal how the networks in PFC is organized at rest and thus pave a new way to better understand the neural mechanism underlying neuroticism/conscientiousness.

## 2 MATERIALS AND METHODS

### 2.1 Participants

Thirty‐five college students were recruited from the University of Macau campus. The protocol was approved by the Institutional Review Board with the University of Macau. All participants were right‐handed with normal or corrected‐to‐normal vision. All participants were required to sign the informed consent documents prior to the experiments. Any participants with histories of neurological or psychiatric disorders were excluded from this study.

### 2.2 Big five personality questionnaire

NEO Five‐Factor Inventory (NEO‐FFT) (McCrae & Costa, 2004, 2007) was adopted to access each participant's personality profile. The NEO‐FFT scale contains 60 questions which measures five personality traits with 12 items in each of five factors: Openness, Conscientiousness, Extraversion, Agreeableness, and Neuroticism (OCEAN). First, individuals with high openness (high‐O) are considered to be more imaginative to art, more intellectually curious and more behaviorally flexible (Costa & McCrae, 1992). Second, individuals with high conscientiousness (high‐C) are more diligent, more well‐organized and well‐determined, and more ambitious compared to those with low‐C. Third, high extraversion (high‐E) denotes a series of traits, which include activity, sociability and inclining to undergo positive emotions such as joy (Costa & McCrae, 1992). In addition, individuals with high agreeableness (high‐A) are cooperative, trusting, and sympathetic whereas the ones with low‐A are callous, cynical and antagonistic (Costa & McCrae, 1992). Finally, individuals with high‐N (Neuroticism) are more likely to experience psychological conditions (Costa & McCrae, 1992).

Interestingly, this scale has been widely used to measure personality traits (McCrae & Costa, 1997). The items of the questionnaire are rated based on a five‐point Likert scale, with 1 and 5 representing “strongly disagree” and “strongly agree,” respectively. The total scores of each personality dimension are ranged from 12 to 60. The descriptive scores of five dimensions for the present work were: O (41.57 ± 5.86), C (45.13 ± 6.60), E (41.13 ± 7.91), A (39.80 ± 5.06), and N (28.30 ± 7.55). In this study, we discovered that conscientiousness was negatively correlated with neuroticism (*r* = −0.53, *p *=* *0.002).

### 2.3 Data acquisition

**Figure 1:**
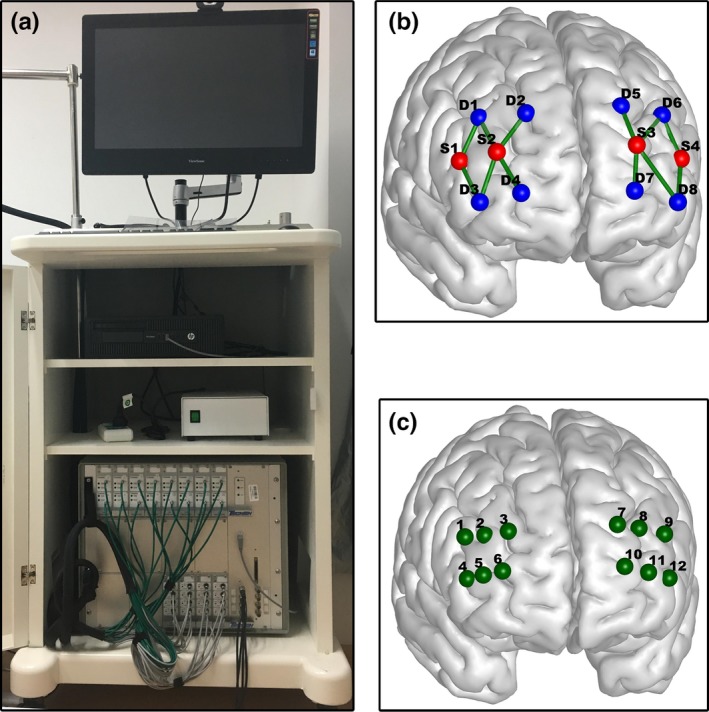
(a) The CW6 fNIRS system. (b) The configuration of the source and detector pairs. The blue and red dots denote the light detectors and laser sources, respectively, and the green lines between each source and each detector represented the channels. (c) The 3D MNI coordinates of the 12 channels

Resting‐state fNIRS data were acquired with TechEn CW6 (Techen Inc., Milford, MA) system as depicted in Figure 1a. A total of four lasers sources with wavelengths at 690 nm and 830 nm and eight light detectors were used to generate 12 channels that covered the left and right lateral prefrontal cortex. The distance between each source and each detector was 3 cm as illustrated in Figure 1b. During the experiment, the participant wore a custom‐built head cap, which was made from plastic and Velcro. The sampling rate was 50 Hz and a total 10‐min resting‐state fNIRS data were acquired. While recording the data, all participants were required to stay still and keep their eyes closed without falling asleep.

After data acquisition, the three‐dimensional (3D) coordinates of each source and detector were measured by using a 3D digitizer (PATRIOT, Polhemus, Colchester, Vermont, USA). The mean 3D coordinates were then imported into NIRS‐SPM (Ye, Tak, Jang, Jung, & Jang, 2009) for spatial registration to generate the layout of optodes and MNI coordinates of each channel (Table 1). The 3D MNI coordinates of 12 channels were displayed in Figure 1c, which were visualized with BrainNet Viewer (Xia, Wang, & He, 2013).

**Table 1:** The mean 3D MNI coordinates and associated brain regions of the 12 channels

Channels	MNI coordinates (*x*,* y*,* z*)			Brodmann area	Probability
CH01	50	43	26	45 ‐ pars triangularis Broca's area	0.88
CH02	42	51	27	46 ‐ Dorsolateral prefrontal cortex	0.74
CH03	32	57	29	46 ‐ Dorsolateral prefrontal cortex	0.86
CH04	50	50	7	46 ‐ Dorsolateral prefrontal cortex	0.80
CH05	44	59	9	46 ‐ Dorsolateral prefrontal cortex; 10 ‐ Frontopolar area	0.56; 0.44
CH06	36	65	11	10 ‐ Frontopolar area	0.89
CH07	−18	62	32	10 ‐ Frontopolar area; 46 ‐ Dorsolateral prefrontal cortex	0.48; 0.36
CH08	−29	56	30	46 ‐ Dorsolateral prefrontal cortex	0.91
CH09	−42	49	27	46 ‐ Dorsolateral prefrontal cortex	0.61
CH10	−20	71	13	10 ‐ Frontopolar area	1
CH11	−32	65	10	10 ‐ Frontopolar area	0.86
CH12	−43	56	7	46 ‐ Dorsolateral prefrontal cortex	0.71

### 2.4 Data processing

Data from five participants were excluded due to extensive body movement. Data processing was further performed for the remaining 30 participants, including 17 females (23.41 ± 2.09 years) and 13 males (23.92 ± 2.25 years). The first 3‐min resting‐state fNIRS recordings were discarded to make the analysis stable (Niu et al., 2012, 2013), whereas only 3‐min resting‐state recordings in the middle section were kept for further analysis. After the band‐pass filter (0.01–0.1 Hz), motion correction and data detrending, the concentration changes of HbO and HbR were generated and the resting‐state functional networks were constructed with FC‐NIRS (Xu et al., 2015).

### 2.5 Small‐world network analysis

The nodes and edges are two key elements to construct a functional connectivity network. In our case, the nodes were defined as the channels and the edges were denoted as the functional connectivity between channels. In this study, an *N* by *N* (*N* = 12, the number of channels in this study) correlation matrix was generated in dlPFC for each participant using parametric Pearson correlation analysis. We then converted the correlation matrix into a binary undirected graph G using the following graph construction:

(1) $$e_{\mathrm{ij}}=\left\{\begin{array}{c} 1,\;\mathrm{if}\;\left|r_{\mathrm{ij}}\right|\geq T\\ 0,\;\mathrm{otherwise} \end{array}\right.$$

If the absolute *r*_*ij*_ exceeded a given threshold *T*, the connection between two nodes was set to 1. Otherwise, it was set to 0. In this study, the threshold *T* was determined by the sparsity (*S*), which was the ratio between the number of actual edges and the maximum possible number of edges in a network. The *S* values were ranged from 0 to 1 with an interval of 0.01 (Niu et al., 2012).

We computed the small‐world network parameters, which include the clustering coefficient (*C*_*p*_), characteristic path length (*L*_*p*_), normalized clustering coefficient (*γ*), and normalized characteristic path length (*λ*). And the efficiency properties of the networks in dlPFC were also generated, which contain the global efficiency (*E*_glob_), local efficiency (*E*_loc_), nodal efficiencies (*E*_nodal_), normalized global efficiency (*γ*), normalized local efficiency (*λ*), and two additional nodal parameters (*K*_nodal_, *N*_bc_). The network characteristics described the ability of information transmission of a network at both the global and local level.

The *C*_*p*_ is defined as the averaged clustering coefficient over all nodes, which measures the local interconnectivity of a network:

(2) $$C_{p}=\frac{1}{N}\sum_{i\in G}\frac{E_{i}}{K_{i\;}\left(K_{i\;}-1\right)/2}\;$$

in which *N* is the number of nodes, and *E*_*i*_ and *K*_*i*_ represent the number of edges and nodes in the subgraph *G*_*i*_ *,* respectively (Rubinov & Sporns, 2010; Watts & Strogatz, 1998).

The *L*_*p*_ is defined as the average of character path length over all nodes, which quantifies the overall routing efficiency of a network,

(3) $$L_{p}=\frac{1}{N}\sum_{i\in G}\frac{\sum_{i\neq j\in G}\min\left|L_{\mathrm{ij}}\right|}{N-1}$$

in which min|*L*_*ij*_| is the shortest path length between the node *i* and node *j* (Rubinov & Sporns, 2010; Watts & Strogatz, 1998).

The normalized clustering coefficient is the ratio between the real and random clustering coefficients: $=\frac{C_{p-\mathrm{real}}}{C_{p-\mathrm{rand}}}$. The normalized characteristic path length is the ratio between the real and random characteristic path length: $\lambda =\frac{L_{p-\mathrm{real}}}{L_{p-\mathrm{rand}}}.$ *C*_*p_*rand_ and *L*_*p_*rand_ denotes, respectively, the averaged clustering coefficient and characteristic path length of 100 matched random networks, which possess the same number of nodes, edges, and degree distribution with the real networks (Maslov & Sneppen, 2002; Sporns & Zwi, 2004). Typically, a small‐word network meets the conditions of γ>1 and *λ* ≈ 1 (Watts & Strogatz, 1998), and therefore, the small‐world scalar *σ* = *λ*/*γ* is larger than 1 (Humphries, Gurney, & Prescott, 2006).

For the network efficiency matrices, *E*_glob_ is the mean of all nodes efficiencies, which is defined as the inversion of harmonic mean of the shortest path length between each node pair (Achard & Bullmore, 2007; Latora & Marchiori, 2001),

(4) $$E_{\mathrm{glob}}=\frac{1}{N}\sum_{i\in G}E_{\mathrm{nodal}}\left(i\right)$$

and

(5) $$E_{\mathrm{nodal}}\left(i\right)=\frac{1}{N-1}\sum_{j\neq i\in G}\frac{1}{\min\left|L_{\mathrm{ij}}\;\right|}$$

in which min |*L*_*ij*_| denotes the shortest path length between the node *i* and node *j*, indicating the capability of parallel information transfer through the whole network. In addition, *E*_loc_ is the average of all local efficiencies for nodes in the subgraph *G*_*i*_ (Achard & Bullmore, 2007; Latora & Marchiori, 2001), which is defined as

(6) $$E_{\mathrm{loc}}=\frac{1}{N}\sum_{i\in G}E_{\mathrm{loc}\_\mathrm{nodal}}\left(i\right)$$

in which *E*_loc_nodal_(*i*) = *E*_glob_(*G*_*i*_). Since the node *i* is not an element of subgraph *G*_*i*_, the local efficiency can also be considered as a measure of the fault tolerance of the network, indicating how well each subgraph exchanges information when the node *i* is eliminated (Achard & Bullmore, 2007).

The normalized global efficiency is the ratio between the real and random global efficiency $\gamma_{E}=\frac{E_{g-\mathrm{real}}}{E_{g-\mathrm{rand}}}$. The normalized local efficient is the ratio between the real and random local efficiency $\lambda_{E}=\frac{E_{l-\mathrm{real}}}{E_{l-\mathrm{rand}}}$. *E*_*g_*rand_ and *E*_*l*_rand_ denotes the averaged global and local efficiency of 100 degree matched random networks (Wang et al., 2009), respectively. Generally, a small‐word network processes $\gamma_{E}>1$ and *λ*_*E*_ ≈ 1, and the small‐wordness scalar *σ*_*E*_ = *γ*_*E*_/*λ*_*E*_ is larger than 1. Further, *K*_nodal_ is the degree index of a node, which is the number of edges generated by a node connecting with other nodes. The larger number indicts that this node connects with more additional nodes. By contrast, *N*_bc_ estimates the influence of a node over information flow with the rest nodes in a network. The network analysis module in software GRETNA (Wang et al., 2015) was used to analyze the network characteristics.

### 2.6 Statistical analysis of the network indicators

To examine the difference between the real network parameters and the ones from associated random network, we computed the *z*‐score with

(7) $$Z\left(x\right)=\frac{x_{\mathrm{real}}-x_{\mathrm{rand}}}{\mathrm{std}\left(x_{\mathrm{rand}}\right)}$$

in which *x*_real_ denotes the network characteristics (*C*_*p*_ *, L*_*p*_ *, E*_glob_ *, E*_loc_). A two‐tailed significance level of 0.05 (*z*‐score <−1.96 or *z*‐score > 1.96) was used to inspect the significant difference.

In order to examine the association between the individuals’ traits and network parameters, we calculated the integral area under curve (AUC) for a network metric *Y*, which was produced over the sparsity threshold ranged from *S*_1_ to *S*_*n*_ with an interval of Δ*S*,

(8) $$Y^{\mathrm{AUC}}=\sum_{k=1}^{n-1}\left[Y\left(S_{k}\right)+Y\left(S_{k+1}\right)\right]\times \Delta S/2.$$

## 3 RESULTS

### 3.1 Small‐world and efficiency characteristics of brain networks in the dLPFC

**Figure 2:**
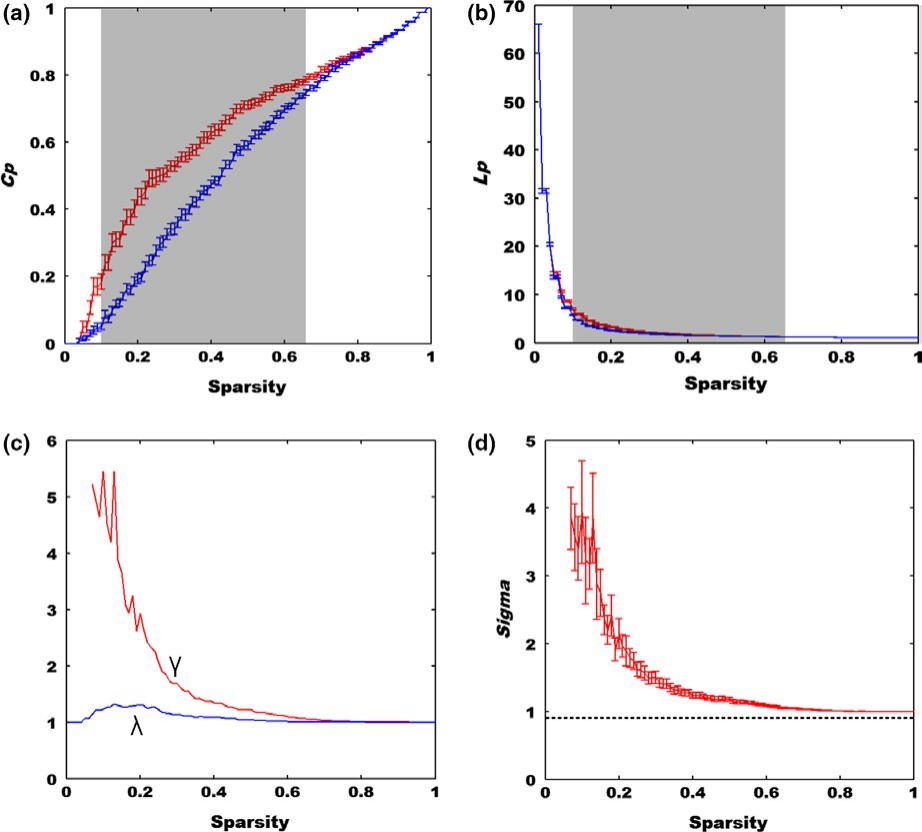
The small‐world properties of real network in dlPFC and matched random network. (a) The cluster coefficient (*C*_*p*_). The shadow window shows that the *C*_*p*_ of real dlPFC network is significantly larger than that of the random network with sparsity from 0.1 to 0.66 (*p *<* *0.05). The red curve denotes the real network of dlPFC while the blue one defines the random network. (b) The characteristic path length (*L*_*p*_). The shadow window shows that the *L*_*p*_ of real dlPFC network is significantly longer than that of the random network (*p *<* *0.05). The red curve denotes the real network of dlPFC, while the blue one defines the random network. (c) Two indictors of the small‐worldness. The red and blue curve denotes the distribution of *γ* and *λ* of real dlPFC network, respectively. *γ *> 1, *λ *≈ 1. (d) Additional indicator of the small‐worldness, *σ *> 1. The network of dlPFC exhibits the small‐world properties

The small‐world analysis results were displayed Figure 2, in which we discovered that the *C*_*p*_ and *L*_*p*_, respectively, increased (Figure 2a) and descended (Figure 2b) with increased sparsity threshold for both the real brain network and random network (Figure 2). Statistical analysis showed that compared to the random network, the real brain network (*C*_*p_*real_) exhibited significant larger *C*_*p*_ for the sparsity *S* ranged from 0.1 to 0.66 (mean *z*‐score = 2.86 ± 0.37). In addition, the *L*_*p*_ of real brain network (*L*_*p_*real_) was also larger than (but numerically approximate to) that of the random network (*L*_*p_*rand_) with *S* ranged from 0.1 to 0.66 (mean z‐score = 8.03 ± 3.27). Further, since the *γ* was larger than 1 and the *λ* approached to 1 (*σ *> 1) (Figure 2c,d), the resting‐state brain networks in dLPFC also exhibited the small‐world properties.

Accordingly, we also calculated the *C*_*p*_ and *L*_*p*_ based on HbR and HbT (Total hemoglobin: the sum of HbO and HbR) recordings (Supporting Information Figures S1 and S2). The results showed that the networks of HbR and HbT also manifested small‐world properties. Compared to that of random matched networks, the *C*_*p*_ of brain networks in dlPFC was larger with sparsity between 0.05 and 0.62 for HbR and 0.07–0.62 for HbT. By contrast, the *L*_*p*_ of HbR or HbT measures didn't exhibit obvious difference as compared to that from the random network.

**Figure 3:**
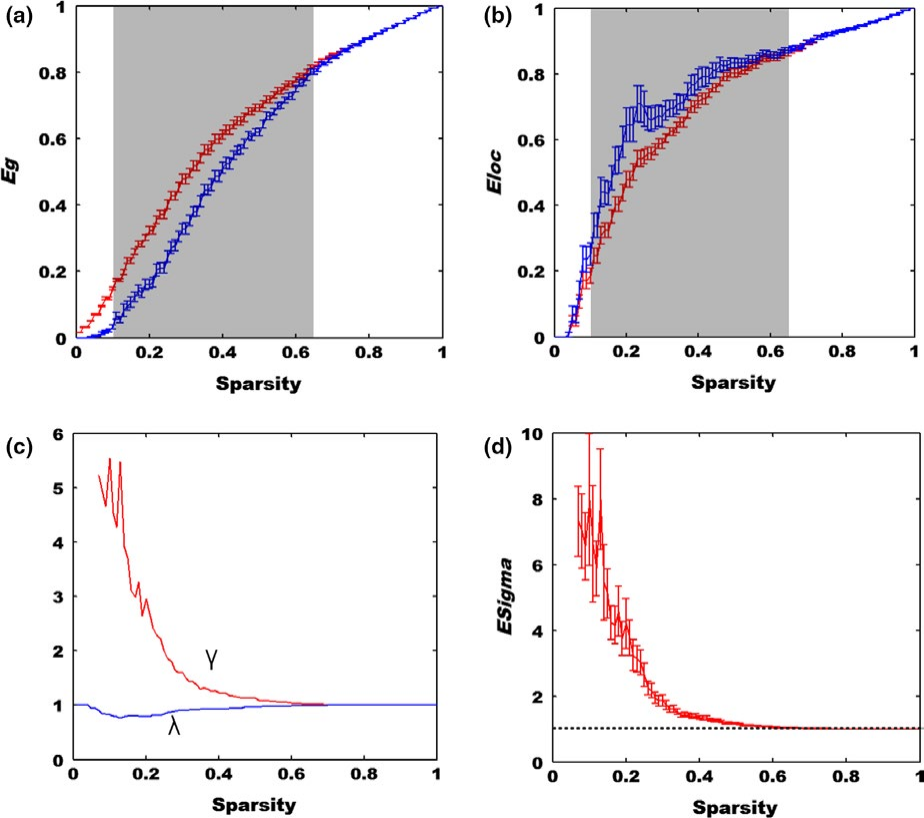
The global and local efficiency of real brain network in dlPFC and the matched random network. (a) The global efficiency (*E*_*g*_). The red curve denotes the real network of dlPFC while the blue curve denotes the matched random network. The shadow window represents the *E*_*g*_ of real dlPFC network is significantly higher than that of the random network with sparsity from 0.1 to 0.66 (*p *<* *0.05). (b) The local efficiency (*E*_loc_). The red curve denotes the real network of dlPFC while the blue curve denotes the matched random network. The shadow window shows that the *E*_loc_ of real dlPFC network is significantly smaller than that of the random network with sparsity from 0.1 to 0.65(*p* < 0.05). (c) The blue curve and red curve denotes the *λ* and *γ* of the efficiency, respectively. (d) The red curve represents the sigma of the efficiency in dlPFC

The profiles of global and local efficiency were depicted in Figure 3, in which we discovered that the *E*_glob_ and *E*_loc_ of the two networks (real network and random network) ascended rapidly and approached to one in the end. Compared to the random network, the real brain network exhibited larger global and smaller local efficiency with sparsity between 0.1 and 0.66 for the global one (mean *z*‐score = 6.32 ± 2.20) and 0.1–0.65 for the local one (mean *z*‐score = −2.47 ± 0.54). Meanwhile, the *γ*_*E*_ was greater than one while the *λ*_*E*_ was equal to one (Figure 3c).

Likewise, we computed the *E*_glob_ and *E*_loc_ based on HbR and HbT signals (Supporting Information Figures S3 and S4), in which we discovered that the results were in line with that of HbO measures. The *E*_glob_ and *E*_loc_ of HbR and HbT were greater than those of the matched random network with a broad sparsity range (HbR, 0.07–0.62 for global one and 0.05–0.41 for local one; HbT, 0.08–0.57 for global one and 0.12–0.36 for local one). In addition, the results of *γ*_*E*_, *λ*_*E*_ from HbR and HbT measures showed good agreement with that of HbO measures.

### 3.2 Network hubs in the dlPFC

**Figure 4:**
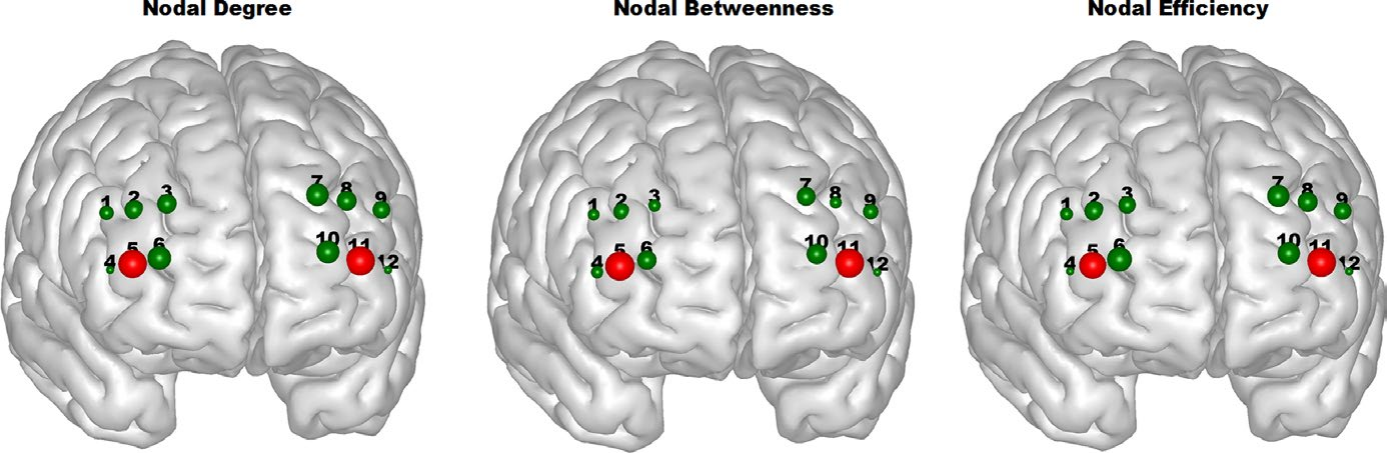
Hubs identified by three nodal indices were displayed in red color. The size of nodes denotes the value of nodal properties

The network hubs were determined by the nodal degree (*K*_nodal_), nodal efficiency (*E*_nodal_), and nodal betweenness (*N*_bc_). If any of the three nodal network properties for a single node was one standard deviation larger than that from the average of all nodes in the network, this node was considered as a hub (Niu et al., 2012). In this study, extensive sparsity (0:0.01:1) values was utilized to construct the functional brain network, in which each of these three nodal parameters was a function of sparsity. In particular, a threshold independent scalar (area under the curve, AUC) was calculated for each nodal parameter (*K*_nodal*,*_ *E*_nodal,_ and *N*_bc_) from each node to determine the network hubs. Consequently, we discovered that Channels 5 and 11 were the hubs determined by the AUCs of *K*_nodal*,*_ *E*_nodal*,*_ and *N*_bc_ (Figure 4).

Similar operations were performed to identify the network hubs by using HbR and HbT signals. Intriguingly, HbR measures showed that channels 2, 3, and 11 were the identified hubs (Supporting Information Figure S5) while HbT measures exhibited that channel 11 was the hub (Supporting Information Figure S6).

### 3.3 Correlation analysis

**Figure 5:**
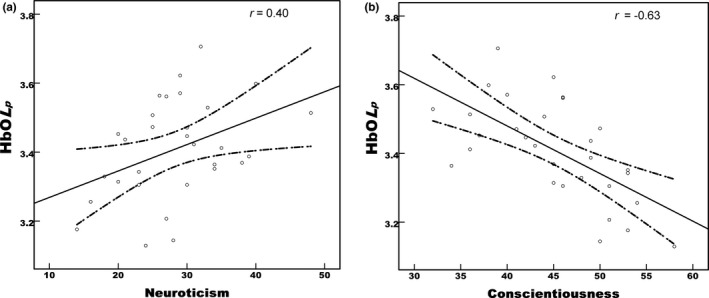
The correlation between the two personality traits and *Lp* of dlPFC. (a) Neuroticism was positively related with the *Lp* (*r *=* *0.40, *p *<* *0.05). (b) Conscientiousness was negatively related with the *Lp* (*r *=* *−0.63, *p *<* *0.001)

In order to explore the individual difference in relationship between the personality traits and topological characteristics of brain networks, the correlation analysis was conducted between the small world properties (*C*_*p*_, *L*_*p,*_ *E*_glob_ and *E*_loc_) and NEO FFT scores. We discovered that conscientiousness (*r *=* *−0.63) showed negative correlation with the *L*_*p*_, whereas neuroticism (*r *=* *0.40) was positively correlated with the *L*_*p*_ (Table 2, Figure 5).

**Table 2:** The correlation coefficients between the small world properties and five personality factors

	HbO*C*_*p*_	HbO*L*_*p*_	HbO*E*_*g*_	HbO *E*_loc_
Neuroticism	0.22	0.40*	−0.20	0.16
Extraversion	−0.04	−0.18	−0.06	−0.002
Openness	0.09	−0.21	−0.003	0.07
Agreeable	−0.02	−0.12	0.13	0.01
Conscientious	−0.23	−0.63**	0.29	−0.15

*p <* *0.05; **p <* *0.01.

## 4 DISCUSSION

In this study, we explored the relationship between individuals’ variations in personality traits and the topological characteristics of resting‐state functional networks in dlPFC using fNIRS. Importantly, the resting‐state networks in dlPFC exhibited the small‐world properties, in which we discovered the clustering coefficient was much higher than that from the random network (Figure 2). These findings were in line with reports from previous studies (Fekete, Beacher, Cha, Rubin, & Mujica‐Parodi, 2014; Niu et al., 2012). In addition, we also discovered that rich hubs across HbO, HbR, or HbT measures were identified for the brain networks in dlPFC (Figure 4, Supporting Information Figures S5 and S6). Further, positive correlations between the neuroticism and characteristic path lengths of HbO and HbT measures were revealed, whereas conscientiousness exhibited negative relationship with the characteristic path length of HbO recordings (Figure 5). Interestingly, little studies have been performed to examine the resting‐state functional brain networks by using fNIRS data and graph theory. To the best of our knowledge, this is the first study that used resting‐state fNIRS data to examine the relationship between the small‐world networks properties and personality traits in the dlPFC. Therefore, our study provided solid evidences that effective neuromarkers can be extracted from the resting‐sate fNIRS data.

The small‐world network was first proposed in 1998 (Watts & Strogatz, 1998), which generally exhibits a higher clustering coefficient and almost the same characteristic path length as compared to a random network (Watts & Strogatz, 1998). These characteristics enable the information to flow more efficiently, which were also evidenced by our global and local efficiency analyses (Figure 3). For example, we discovered that our resting‐state brain functional network manifested a higher global and local efficiency than the random network, which showed good agreement with previous fMRI and fNIRS findings (Achard et al., 2006; Fekete et al., 2014; Niu et al., 2012). In particular, our resting‐state fNIRS results demonstrated that the brain network in local dlPFC is also organized economically and efficiently just as the whole brain does. More importantly, our results further indicated that the small‐world architecture is a ubiquitous organization of our brain regardless of different imaging methods (Niu et al., 2012). Besides, we also inspected the node hubs of resting‐state functional brain networks in dlPFC. Interestingly, previous work showed that the brain hubs play an essential role in information integration underpinning numerous aspects of complex cognitive functions (van den Heuvel & Sporns, 2013). In this study, three nodal characteristics were used to identify brain hubs in dlPFC using resting‐state fNIRS recordings. We discovered that channels 2, 3, 5, and 11 were the identified network hubs based on three measures (HbO, HbR, and HbT). When the analysis results from the three measures were combined together, we observed that channels 5 and 11 played a crucial role for the network organization in dlPFC, which were symmetrically located in the right and left hemispheres, respectively. In particular, the identified hubs demonstrated that each part of left and right dlPFC was involved in the information processing framework on brain cognition.

In addition, our results showed that neuroticism was positively correlated with the shortest path length from HbO and HbT measures while conscientious was inversely associated with the characteristic path length of HbO measures. Neuroticism is related to emotion stability and vulnerability and negative affection. A high score of this factor represents one of the most common psychiatric conditions (Costa & McCrae, 1992). In addition, our results also demonstrated that individuals who are vulnerable to neurosis or who are experiencing more negative feelings exhibit the less efficiency and the longest path length in processing information in dlPFC. Interestingly, previous clinical study had validated that the depression patients exhibited the long characteristic path length (Meng et al., 2013). Additional reported work further demonstrated that neuroticism was positively related with brain activation in dlPFC during viewing negative pictures (Canli et al., 2001) or emotion regulation (Harenski et al., 2009).

By contrast, conscientiousness is a dimension that contrasts scrupulous, well‐organized, and diligent people with lax, disorganized, and lackadaisical individuals (Costa & McCrae, 1992). Those who score high for this factor are considered to be more self‐controlled and motivated. Conscientiousness is also associated with academic and vocational success (Judge, Higgins, Thoresen, & Barrick, 1999; Noftle & Robins, 2007; O'Connor & Paunonen, 2007). Our results showed that conscientiousness was negatively correlated with the *L*_*p*_ in dlPFC, which plays an essential role in executive function. The higher conscientiousness scores are, the shorter characteristic path length is. Meanwhile, our results suggested that individuals with well‐organized, persistence and self‐discipline tend to process information more efficiently and economically, which showed good agreement with previous reports. For example, the relationship between the *L*_*p*_ in dlPFC and neuroticism/conscientiousness was also highlighted in previous studies, in which they discovered that neuroticism and conscientiousness, respectively, constrained and facilitated neuroplastic responses within the working memory networks, which includes the dlPFC, parietal, and anterior cingulate cortex (Dima, Friston, Stephan, & Frangou, 2015). Interestingly, previous studies also demonstrated that compromised small‐world properties in dlPFC was associated with reduced levels of effort control, which was related with psychopathology in young children (Fekete et al., 2014).

In summary, our study showed that fNIRS can be an efficient technique to explore the brain network of special atypical populations at rest, which might not be appropriate for them such as neonates, infants, and claustrophobia to participate in fMRI tests due to its narrow space and restrained positions. Several limitations of this study should be mentioned here. First, although the validity and reliability of NEO‐FFI is stable, we cannot fully exclude subjective judgment because it is a self‐report measurement (Dima et al., 2015). Second, only the dlPFC is involved in this study, which somehow ignores the influence from other brain regions. Third, it should be pointed out that the relatively small sample sizes might affect the accuracy of present analysis results. Nevertheless, our results demonstrated that neuroticism and conscientiousness, respectively, are positively and negatively correlated with the *L*_*p*_ in dlPFC, indicating that resting‐state fNIRS can be a promise tool for early detection of brain disorders.

## CONFLICT OF INTEREST

The authors declare no conflict of interests.
