# Supplementary material for: Neuroticism and conscientiousness respectively positively and negatively correlated with the network characteristic path length in dorsal lateral prefrontal cortex: A resting‐state fNIRS study
Source: Brain Behav. 2018 Jul 27;8(9):e01074. doi: 10.1002/brb3.1074 (PMC6160652; doi:10.1002/brb3.1074)
Supplement: Supplementary file 1 [file BRB3-8-e01074-s001.docx]

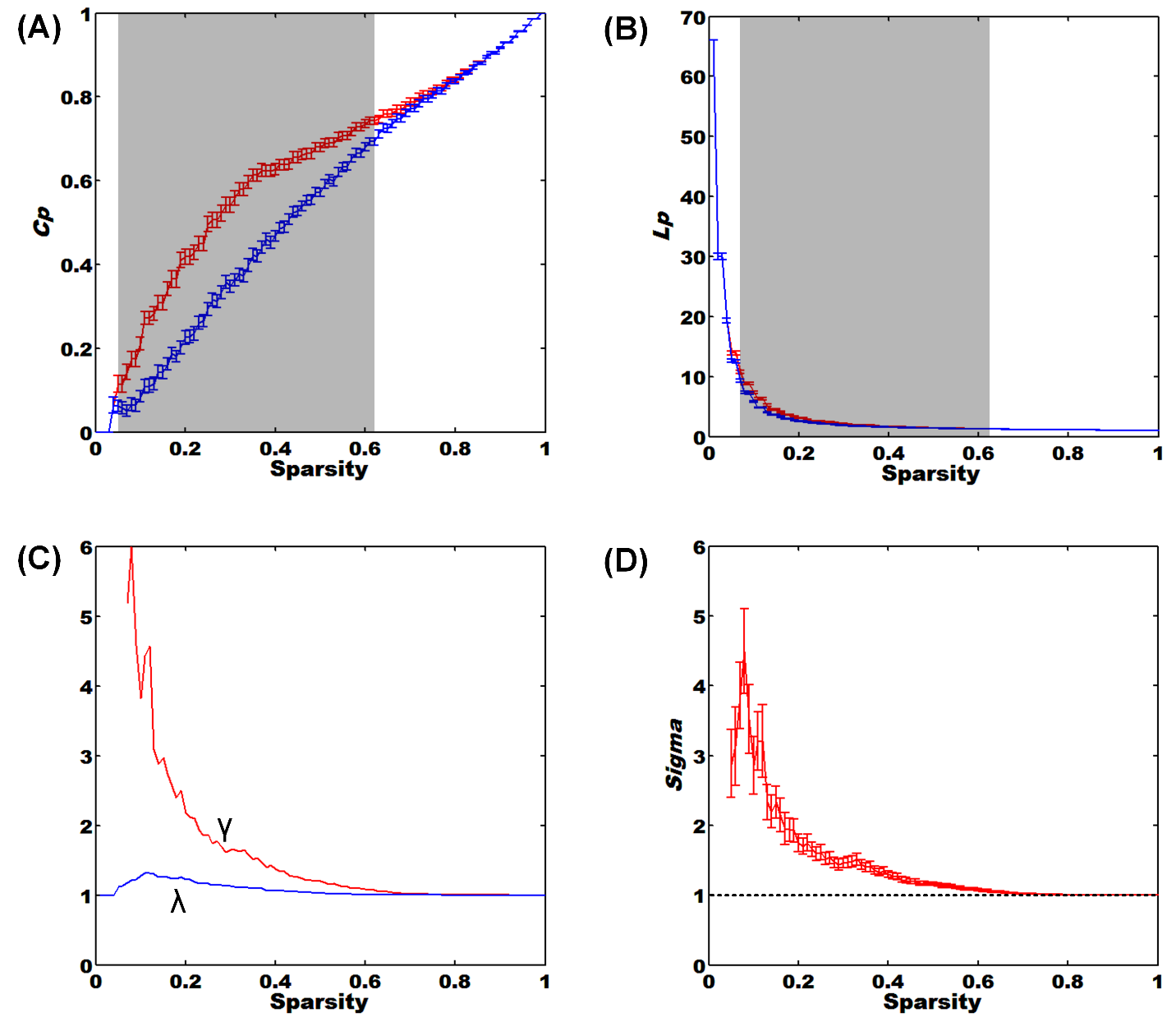


**Fig. S1** **The value of small world characters of HbR.** (**A) and (B):** The blue lines denote matched random network while the red lines represent the real brain network. The grey arears represent the significant difference between the real brain functional network and the random matched network. **(C):** The blue line and the red line denote the value of λ and γ, respectively. **(D):** The red line represents the value of sigma of the real brain network.


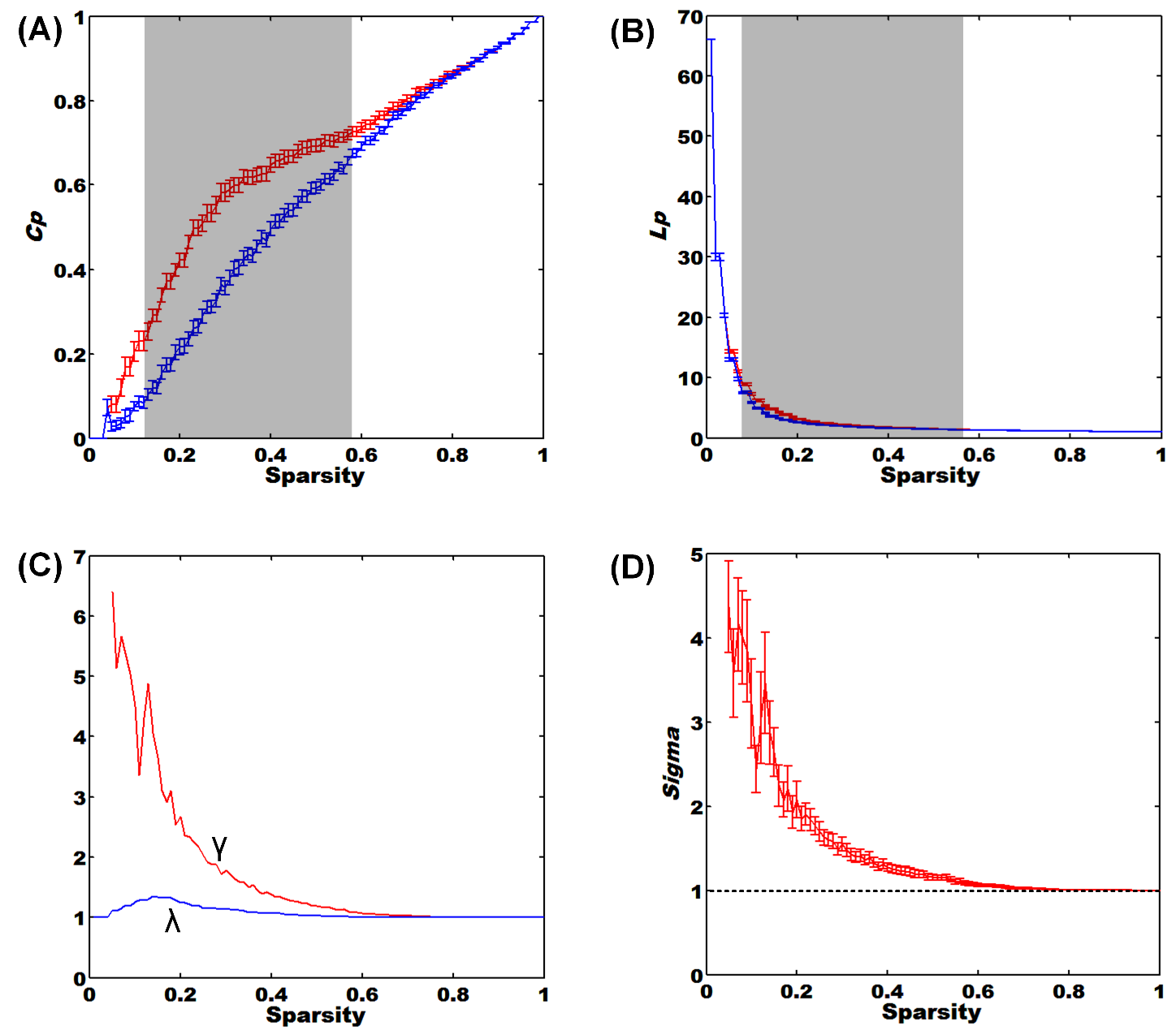


**Fig. S2 The value of small world characters of HbT.** (**A) and (B):** The blue lines denote matched random network while the red lines represent the real brain network. The grey arears represent the significant difference between the real brain functional network and the random matched network. **(C):** The blue line and the red line denote the value of λ and γ, respectively. **(D):** The red line represents the value of sigma of the real brain network.


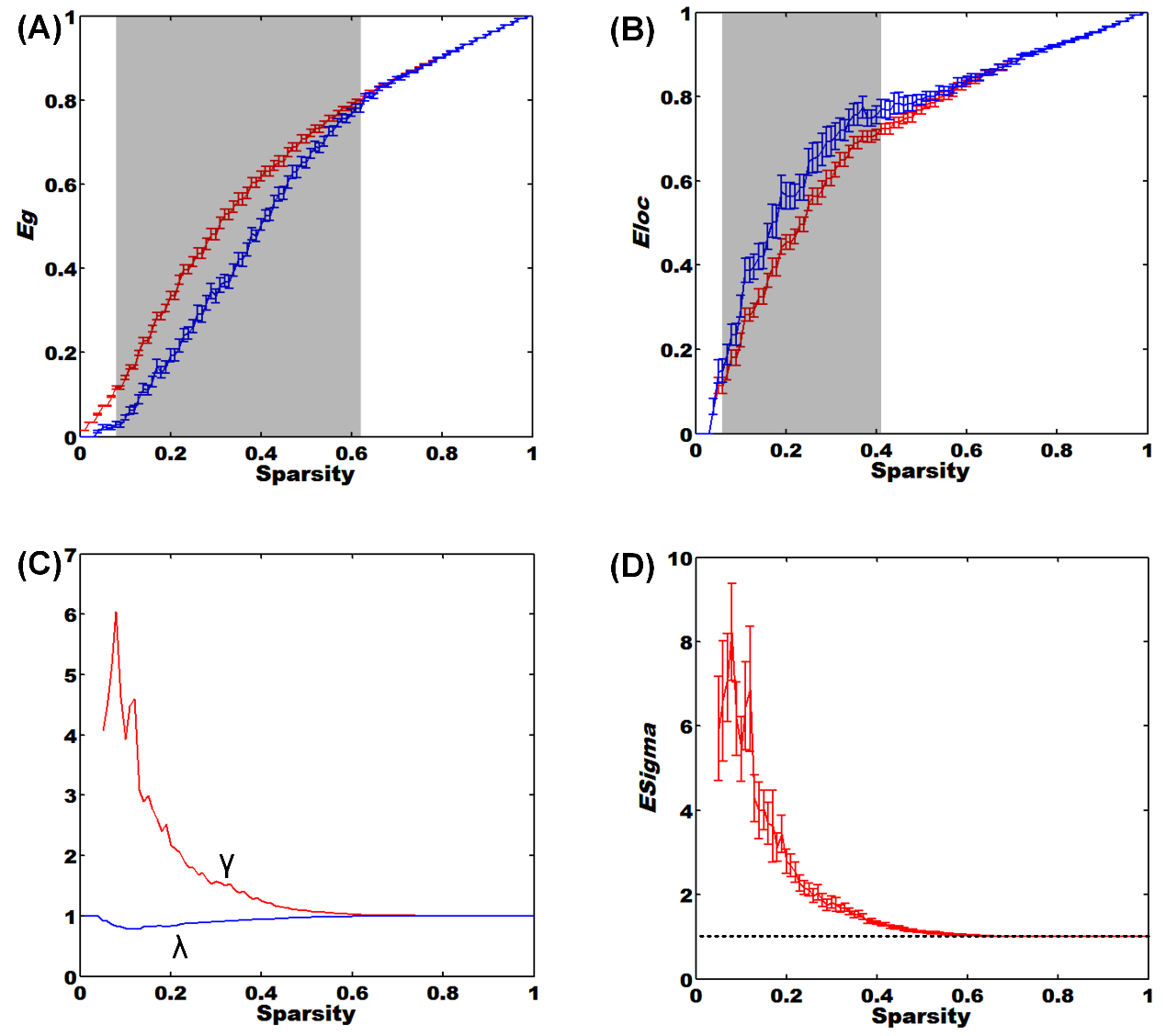


**Fig. S3 The value of global and local efficiency of HbR.** **(A) and (B):** The blue lines denote matched random network while the red lines represent the real brain network. The grey arears represent the significant difference between real brain functional network and the random matched network. **(C):** The blue line and the red line denote the value of λ and γ, respectively. **(D):** The red line represents the value of sigma of the real brain network.


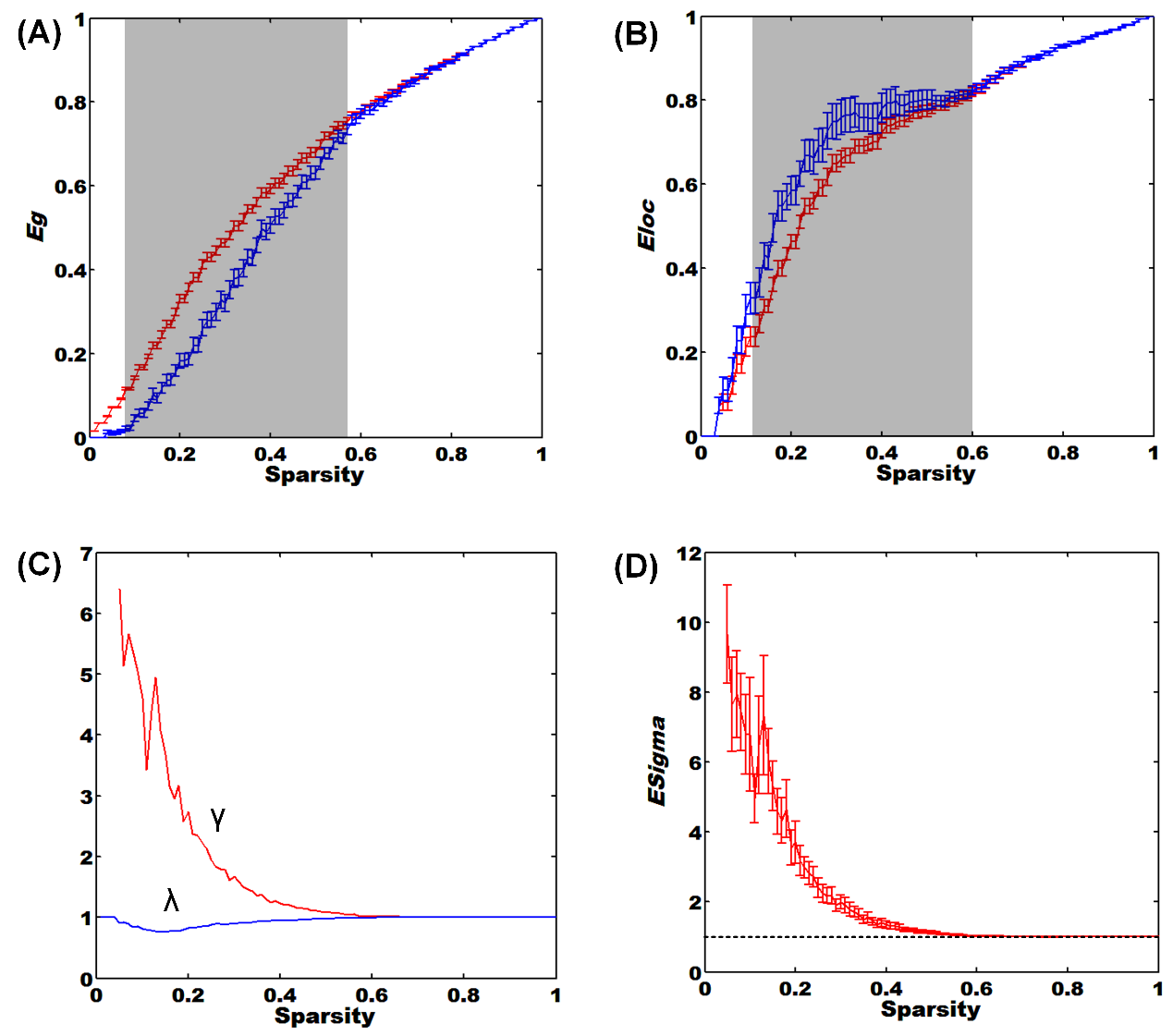


**Fig. S4 The value of global and local efficiency of HbT.** **(A) and (B):** The blue lines denote matched random network while the red lines represent the real brain network. The grey arears represent the significant difference between real brain functional network and the random matched network. **(C):** The blue line and the red line denote the value of λ and γ, respectively. **(D):** The red line represents the value of sigma of the real brain network.


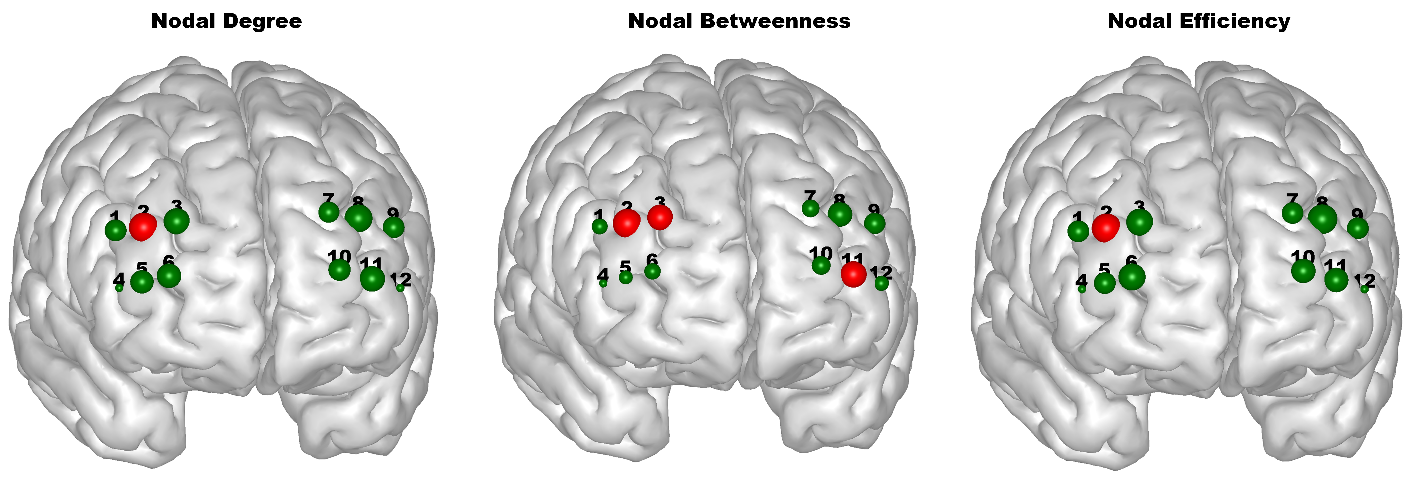


**Fig. S5 The hubs of HbR identified by three nodal indices were illustrated in red color.** The size of the nodes denotes the value of the according nodal properties.


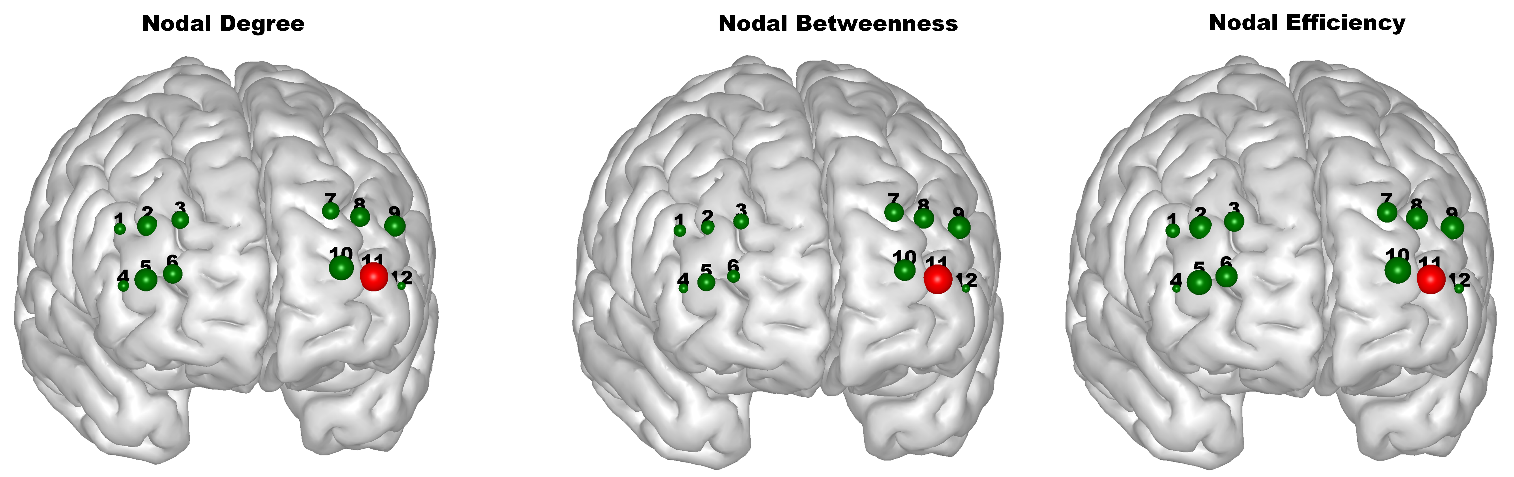


**Fig. S6 The hubs of HbT identified by three nodal indices were illustrated in red color.** The size of the nodes denotes the value of the according nodal properties.
